# Association of Germline *CHEK2* Gene Variants with Risk and Prognosis of Non-Hodgkin Lymphoma

**DOI:** 10.1371/journal.pone.0140819

**Published:** 2015-10-27

**Authors:** Ondrej Havranek, Petra Kleiblova, Jan Hojny, Filip Lhota, Pavel Soucek, Marek Trneny, Zdenek Kleibl

**Affiliations:** 1 First Department of Medicine–Department of Hematology, First Faculty of Medicine, Charles University in Prague and General University Hospital in Prague, Prague, Czech Republic; 2 Institute of Biochemistry and Experimental Oncology, First Faculty of Medicine, Charles University in Prague, Prague, Czech Republic; 3 Toxicogenomics Unit, National Institute of Public Health, Prague, Czech Republic; CNR, ITALY

## Abstract

The checkpoint kinase 2 gene (*CHEK2*) codes for the CHK2 protein, an important mediator of the DNA damage response pathway. The *CHEK2* gene has been recognized as a multi-cancer susceptibility gene; however, its role in non-Hodgkin lymphoma (NHL) remains unclear. We performed mutation analysis of the entire *CHEK2* coding sequence in 340 NHL patients using denaturing high-performance liquid chromatography (DHPLC) and multiplex ligation-dependent probe amplification (MLPA). Identified hereditary variants were genotyped in 445 non-cancer controls. The influence of *CHEK2* variants on disease risk was statistically evaluated. Identified *CHEK2* germline variants included four truncating mutations (found in five patients and no control; P = 0.02) and nine missense variants (found in 21 patients and 12 controls; P = 0.02). Carriers of non-synonymous variants had an increased risk of NHL development [odds ratio (OR) 2.86; 95% confidence interval (CI) 1.42–5.79] and an unfavorable prognosis [hazard ratio (HR) of progression-free survival (PFS) 2.1; 95% CI 1.12–4.05]. In contrast, the most frequent intronic variant c.319+43dupA (identified in 22% of patients and 31% of controls) was associated with a decreased NHL risk (OR = 0.62; 95% CI 0.45–0.86), but its positive prognostic effect was limited to NHL patients with diffuse large B-cell lymphoma (DLBCL) treated by conventional chemotherapy without rituximab (HR-PFS 0.4; 94% CI 0.17–0.74). Our results show that germ-line *CHEK2* mutations affecting protein coding sequence confer a moderately-increased risk of NHL, they are associated with an unfavorable NHL prognosis, and they may represent a valuable predictive biomarker for patients with DLBCL.

## Introduction

Non-Hodgkin lymphomas (NHL) are a heterogeneous group of lymphoid malignancies that rank seventh in the causes of cancer-related deaths worldwide, with higher incidence in developed countries [1]. The annual incidence rate of NHL in the Czech Republic is approximately 12 per 100 000 inhabitants [2]. The majority of NHL cases arise sporadically, but various observations (familial clustering, and increased risk of NHL in monozygotic twins or other relatives of patients diagnosed with hematological malignancy) indicate that NHL development is affected by genetic factors that are only partially understood [3–6].

**Fig 1:**
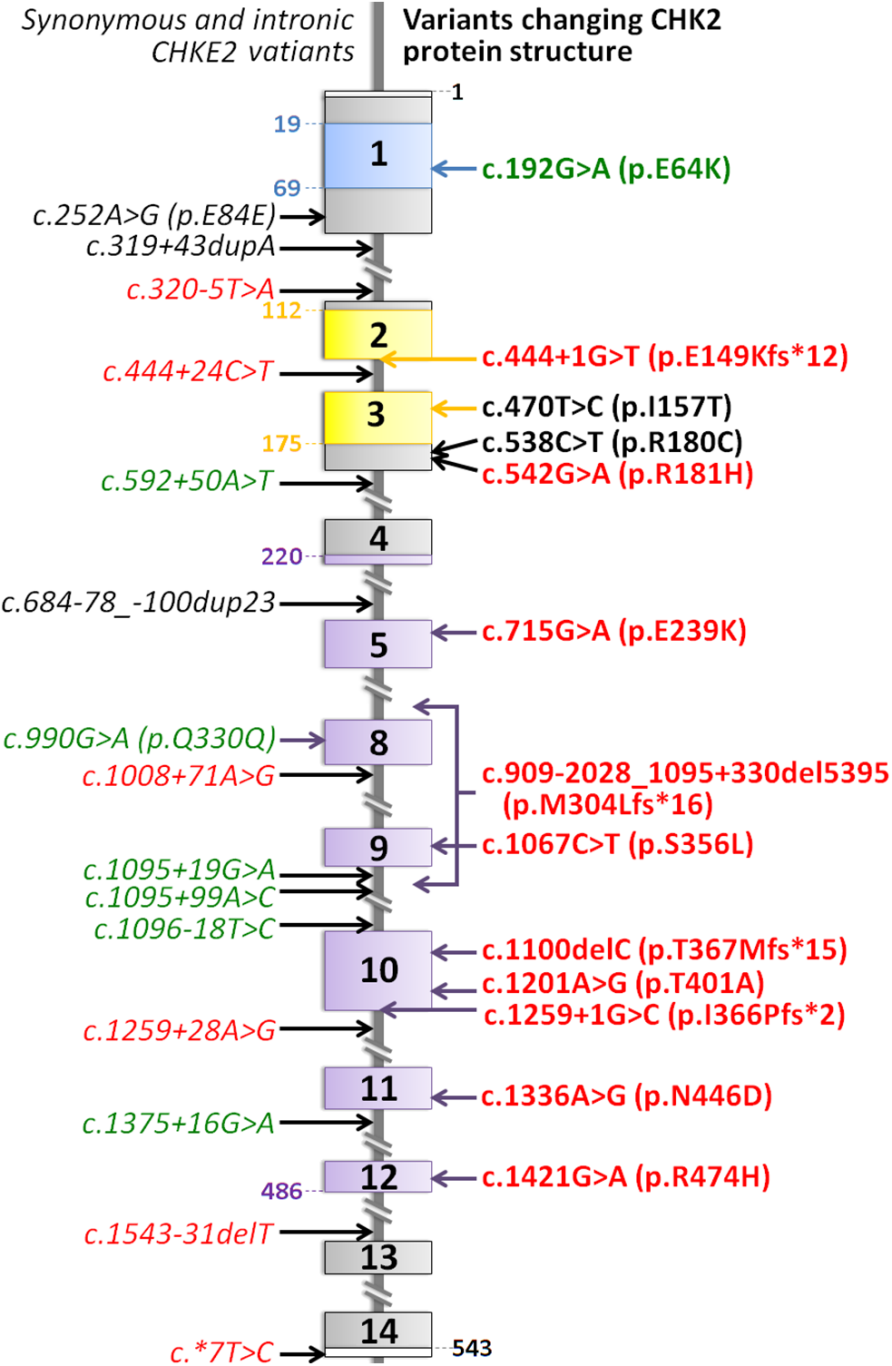
A schematic diagram showing individual coding exons and flanking intronic sequences affected by the identified *CHEK2* sequence variants. The most important structural/functional domains of CHK2 kinase are depicted by color bars [SQ/TQ domain (amino acid (aa) 19–69) in blue, FHA domain (aa 112–175) in yellow, and kinase domain (aa 220–486) in violet]. The left-hand side shows synonymous and intronic *CHEK2* variants (italicized) while the right-hand side shows CHK2 protein structure-altering variants (frame-shift and missense) that were described in the NHL patients group (in red), controls (green) or in both populations (in black).

The checkpoint kinase 2 (*CHEK2*) gene (OMIM 604373) coding for CHK2 protein has been reported as a moderate-penetrance, multi-organ cancer susceptibility gene whose alterations increase the risk of different malignancies including breast, colorectal or prostate cancers [7]. CHK2 is a nuclear serine/threonine kinase contributing to the ATM-CHK2-p53 cascade, a part of the DNA damage response (DDR) system. Human CHK2 consists of three distinct functional domains, a N-terminal SQ/TQ cluster domain (SCD), a forkhead-associated domain (FHA), and a C-terminal Ser/Thr kinase domain (reviewed in [8]; Fig 1). The CHK2 kinase is activated by ATM-mediated phosphorylation [9]. Activated CHK2 consequently phosphorylates several effector proteins involved in regulation of the cell cycle and apoptosis upon DDR. The DDR and DNA repair systems represent a critical anti-cancer barrier activated by various cancer-promoting stimuli in precancerous lesions [10–12]. An impairment of this barrier caused by somatic or inherited alterations in genes coding for proteins involved in DDR or DNA repair induces genomic instability, one of the hallmarks of cancer cells [13]. Frequent occurrence of chromosomal rearrangements in NHL suggests that DDR and DNA repair may be constitutively deficient in NHL patients [14]. Moreover, it has been reported that inter-individual variability in the DDR system could modify the efficacy of chemotherapy during the treatment of hematological malignancies [15]. The relevance of *CHEK2* alterations to NHL remains unclear; however, a known increased risk of lymphoid malignancies (including childhood NHL) in patients suffering from ataxia-telangiectasia (a rare inherited cancer-prone syndrome caused by germ-line mutations in the *ATM* gene coding an upstream CHK2 activator) implicates *CHEK2* as a gene in which hereditary alterations may modify the risk of NHL development [16, 17]. In this study, we performed a mutation analysis of the entire coding sequence of *CHEK2* to establish the influence of inherited *CHEK2* alterations on the risk and course of NHL.

## Materials and Methods

### Study populations

Five hundred and twenty-six NHL patients treated with first-line therapy were enrolled in the study at the First Department of Medicine–Department of Hematology, First Faculty of Medicine, Charles University in Prague and General University Hospital in Prague. The only enrollment criterion was a histologically confirmed diagnosis of NHL according to the WHO Classification of Tumors of Hematopoietic and Lymphoid Tissues. The initial analysis was performed in a group of 340 unselected NHL patients enrolled between May 2000 and June 2008 (Table 1). A validation group of 186 consecutive patients with DLBCL enrolled between July 2008 and May 2011 (Table 1) was used for an independent analysis of the c.319+43dupA variant that influenced the NHL prognosis in analysis of DLBCL subgroup from 340 unselected NHL patients. All clinical data were retrieved from the register of the Czech Lymphoma Study Group (www.lymphoma.cz) and from the patients’ medical records. The control population consisted of 445 randomly selected non-cancer individuals. The inclusion criteria and characteristics of the control group consisting of 445 randomly selected non-cancer individuals were described previously [18]. All patients and controls were of Caucasian origin from the same geographical area. All participating subjects signed informed consent with genetic testing approved by the local ethics committee of the General University Hospital in Prague no. 23/07.

**Table 1:** Clinical characteristics of NHL patients (N = 340) and DLBCL patients from the validation group (N = 186).

Histological subtype	DLBCL	FL	MCL	MALT	B SLL	Other	All NHLcases	Validation DLBCL
**Patients**; N(% of all NHL)	180 (52.9)	71 (20.9)	19 (5.6)	16 (4.7)	11 (3.2)	43 (13.5)	340(100.0)	186
**Age at diagnosis**; median of years (range)	58.7(20.9–83.4)	57.3(28.4–79.4)	63.1(46.6–81.9)	69.9(46.1–84.3)	65.8(47.8–84.5)	58.7(17.4–86.4)	59.6(17.4–86.4)	65.0(20.1–97.3)
**Male**; N(%)	101 (56.1)	36 (50.7)	14 (73.7)	7 (43.8)	8 (72.7)	21 (48.8)	187 (55.0)	92 (49.5)
**Female**; N(%)	79 (43.9)	35 (49.3)	5 (26.3)	9 (56.2)	3 (27.3)	22 (51.2)	153 (45.0)	94 (50.5)
**Clinical stage**; N (%)								
I	45 (25.7)	7 (10.1)	0	5 (31.3)	0	6 (15.8)	63 (19.4)	33 (18.4)
II	37 (21.1)	12 (17.4)	0	2 (12.5)	0	6 (15.8)	57 (17.5)	34 (19.2)
III	22 (12.6)	16 (23.2)	2 (11.8)	2 (12.5)	0	4 (10.5)	46 (14.2)	24 (13.6)
IV	71 (40.6)	34 (49.3)	15 (88.2)	7 (43.8)	11 (100.0)	22 (57.9)	160 (47.1)	86 (48.6)
Missing	5 (2.8)	2 (2.8)	2 (12.5)	0	0	5 (11.6)	14 (4.1)	9 (4.8)
**IPI**; N (%)								
Low	64 (36.6)	38 (55.9)	2 (11.8)	5 (31.3)	0	13 (38.2)	122 (38.1)	41 (24.6)
Low intermediate	56 (32.0)	17 (25.0)	6 (35.3)	4 (25.0)	5 (50.0)	5 (14.7)	93 (29.1)	35 (21.0)
High intermediate	26 (14.9)	9 (13.2)	5 (29.4)	5 (31.3)	4 (40.0)	7 (20.6)	56 (17.5)	36 (21.6)
High	29 (16.6)	4 (5.9)	4 (23.5)	2 (12.5)	1 (10.0)	9 (26.5)	49 (15.3)	55 (32.9)
Missing	5 (2.8)	3 (4.2)	2 (10.5)	0	1 (9.1)	9 (20.9)	20 (5.9)	19 (10.2)
**FLIPI**; N (%)								
Low	-	31 (46.3)	-	-	-	-	-	-
Intermediate	-	21 (31.3)	-	-	-	-	-	-
High	-	15 (22.4)	-	-	-	-	-	-
Missing	-	4 (5.6)	-	-	-	-	-	-

*DLBCL*—diffuse large B-cell lymphoma; *FL*—follicular lymphoma; *MCL*—mantle cell lymphoma; *MALT*—mucosa-associated lymphoid tissue lymphoma; *B SLL*—small lymphocytic lymphoma; *IPI*—international prognostic index; *FLIPI*—follicular lymphoma prognostic index.

### Mutation analysis of the *CHEK2* gene

Genomic DNA was extracted from the peripheral blood of patients and controls by standard procedures. All coding *CHEK2* exons with intron-exonic boundaries were PCR-amplified in 14 fragments. To overcome the presence of numerous pseudogenes, a nested PCR was performed for exons 10–14 as described previously [19, 20]. PCR-amplified fragments were analyzed by denaturing high-performance liquid chromatography (DHPLC; WAVE3500; Transgenomic). Primers, PCR and DHPLC conditions are listed in S1 Table. Samples with aberrant elution profiles on DHPLC were sequenced from independent amplifications (ABI 3130; Life Technologies). The frequencies of identified alterations were estimated in the control group by the same method.

### Analysis of *CHEK2* intragenic rearrangements

A multiplex ligation-dependent probe amplification (MLPA) kit (SALSA MLPA kit P190 CHEK2; MRC-Holland, www.mrc-holland.com) was used according to the manufacturer’s instructions. The analysis of *CHEK2* 5395 bp deletion (c.909-2028_1095+330del5395; M304Lfs*16) in MLPA-positive samples and controls was performed by a PCR-based method described previously [21, 22].

### Analysis of variants affecting *CHEK2* pre-mRNA splicing

In order to evaluate the influence of the identified variants affecting the conservative splicing site (at the start of intron 2 and 10, respectively) on mRNA splicing, peripheral lymphocyte-derived RNA was extracted in particular mutations carriers during their regular follow-up and was reverse transcribed into cDNA using SuperScript III reverse transcriptase (Life Technologies), as described previously [23]. The amplicons covering exons 1–3 (sized 473 bp) and exons 9–11 (sized 314 bp) were amplified using primers 5’-ATATCCAGCTCCTCTACCAGC and 5’-CTGCTTAGTGACAGTGCAATTTCAG, and 5’-CATGAAAACGGTATTATACACCGTGAC and 5’-CCACTGGTGATCTGATCCTTCAG, respectively, and sequenced using the same primers on ABI 3130 (Life Technologies).

### Nomenclature of *CHEK2* alterations and in silico analyses

The nomenclature of *CHEK2* alterations reflects the Human Genome Variation Society guidelines (http://www.hgvs.org/mutnomen/) using NCBI *CHEK2* Reference Sequences NG_008150.1 (gene) and NM_007194.3 (mRNA). The first coding exon of *CHEK2* was designated here as exon 1 in line with the convention maintained in relevant literature.

The biological significance of missense variants was evaluated using the freely available web-based program Align-GVGD [24], SIFT [25], PolyPhen [26] and MutationTaster [27]. The effects of intronic variants on splicing were evaluated using the Alamut (Interactive Biosoftware) application [28].

### Statistical analyses

The two-sided Fisher's exact test was used for the evaluation of the differences between frequencies of alterations in the analyzed groups and subgroups. Odds ratios (OR) were calculated from 2 x 2 contingency tables. Differences in the patients’ clinical characteristics were tested by nonparametric Wilcoxon or the Kruskal-Wallis tests or Spearman rank correlation. A survival analysis was performed using the Kaplan-Meier method; the differences between survival curves were evaluated by Wilcoxon and Log-rank tests and the hazard ratio (HR) was calculated using the Cox proportional hazard model. Survival data were available for 330 NHL patients from the initial group and all 186 patients from the DLBCL validation group with a median follow-up of patients alive at the last contact at 7.94 and 2.77 years, respectively. Progression-free survival (PFS) was defined as an interval from the date of diagnosis to the date of progression, relapse or death from any cause or the last follow-up date after the first-line treatment. Overall survival (OS) was defined as an interval from the date of diagnosis to the date of death from any cause or the last follow-up date. All analyses were performed using Statistica *v*.9.0 (StatSoft) or NCSS *v*.2007 (NCSS).

## Results

### Characterization of *CHEK2* alterations and their impact on the risk of NHL development

We performed a germline sequence analysis of all known protein-coding exons of the *CHEK2* gene in 340 NHL patients. We identified four different mutations resulting in frame-shift and introduction of premature stop codons (truncations) and nine different missense variants (Fig 1 and Table 2) in the patients’ group (the genotype data of 340 analyzed NHL patients are available in S5 Table). The overall frequency of these alterations changing the amino acid sequence of the wt CHK2 protein was significantly higher in NHL patients (25/340; 7.4%) compared to controls (12/445, 2.7%; *P* = 0.003). Truncating variants were identified only in NHL patients (*P* = 0.02; Table 2). Two of them, c.1100delC and 5395 del, lead directly to a frame-shift and premature termination of translation. The other two truncating alterations, c.444+1G>T and c.1259+1G>C, were located in consensus splice donor sites; their effect on *CHEK2* pre-mRNA splicing, resulting in frame shifts and premature termination of translation, was confirmed by sequencing of mRNA (resp. cDNA; S1 and S2 Figs). The MLPA analysis revealed only c.1100delC and 5395 del mutations (S3 Fig). The frequency of the most common missense variant c.470T>C (p.I157T) did not differ significantly between NHL patients and controls (Table 2). Analysis of c.470T>C and another eight missense mutations (seven found in the NHL group and two in controls) by mutation impact prediction software was inconclusive as to likely functional consequences (S2 Table), with the exception of p.R181H and p.N446D being consistently classified as neutral polymorphisms. Besides the 13 different mutations affecting the CHK2 protein sequence (Fig 1 and Table 2), another 15 variants including silent mutations and variants located deeper in intronic sequences and untranslated (UTR) regions were found (Fig 1 and Table 3). The most frequent variant of *CHEK2* identified in our study was intronic polymorphism c.319+43dupA. Interestingly, its frequency was significantly lower in NHL patients compared with controls: 75/340 (22.1%) vs. 139/445 (31.2%), respectively (*P* = 0.005; Table 3).

**Table 2:** Germline alterations of the *CHEK2* gene changing the CHK2 protein structure identified in NHL patients and controls with their frequencies and related odds ratios (OR).

Part of gene	Alteration; Genomic (protein) change	dbSNP rs number	NHL (N = 340);*N* (%)	Controls (N = 445);*N* (%)	OR (95% CI)	*P* value
**Truncating variants**						
i2	c.444+1G>T (p.E149Kfs*12)	rs121908698	1 (0.3)	0	-	-
i7-i9	c.909-2028_1095+330del5395 (p.M304Lfs*16)	-	2 (0.6)	0	-	-
E10	c.1100delC (p.T367Mfs*15)	-	1 (0.3)	0	-	-
i10	c.1259+1G>C (p.I366Pfs*2)	rs121908707	1 (0.3)	0	-	-
*All truncating alterations*			*5 (1*.*5)*	*0*	*-*	*0*.*02*
**Missense variants**						
E1	c.192G>A (p.E64K)	rs141568342	0	1 (0.2)	-	-
E3	c.470T>C (p.I157T)	rs17879961	14 (4.1)	10 (2.2)	1.87 (0.82–4.26)	0.15
E3	c.538C>T (p.R180C)	rs77130927	1 (0.3)	1 (0.2)	1.31 (0.08–21.03)	1.00
E3	c.542G>A (p.R181H)	rs121908701	1 (0.3)	0	-	-
E5	c.715G>A (p.E239K)	rs121908702	1 (0.3)	0	-	-
E9	c.1067C>T (p.S356L)^a^	rs121908703	1 (0.3)	0	-	-
E10	c.1201A>G (p.T401A) ^a^	rs121908704	1 (0.3)	0	-	-
E11	c.1336A>G (p.N446D)	rs121908705	1 (0.3)	0	-	-
E12	c.1421G>A (p.R474H) ^a^	rs121908706	1 (0.3)	0	-	-
*All missense variants*			*21 (6*.*2)*	*12 (2*.*7)*	*2*.*38 (1*.*15–4*.*90)*	*0*.*02*
*All alterations affecting coding sequence*			*25* ^b^ *(7*.*4)*	*12 (2*.*7)*	*2*.*86 (1*.*42–5*.*79)*	*0*.*003*

^a^ New alterations

^b^ Two alterations of the *CHEK2* coding sequence were identified in one patient (c.470T>C and c.1259+1G>C); *OR*–odds ratio; *CI*–confidence interval.

Note: The nomenclature of *CHEK2* alterations was based on NCBI *CHEK2* Reference Sequences NG_008150.1 (gene) and NM_007194.3 (mRNA).

**Table 3:** Germline intronic and silent alterations in the *CHEK2* gene in NHL patients and controls with their frequencies and related odds ratios (OR).

Part of gene	Alteration	dbSNPrs number	NHL (N = 340);*N* (%)	Controls (N = 445);*N* (%)	OR (95% CI)	*P* value
E1	c.252A>G (p.E84E)	rs1805129	22 (6.5)	28 (6.3)	1.03 (0.58–1.84)	1.00
i1	c.319+43dupA (A/AA)^b^	rs17879991	67 (19.7)	131 (29.4)	0.59 (0.42–0.82)	0.002
i1	c.319+43dupA (AA/AA)^b^	rs17879991	8 (2.4)	8 (1.8)	1.32 (0.49–3.54)	0.62
i1	c.319+43dupA (A/AA+AA/AA)^b^	rs17879991	75 (22.1)	139 (31.2)	0.62 (0.45–0.86)	0.005
i1	c.320-5T>A	rs121908700	1 (0.3)	0	-	-
i2	c.444+24C>T	rs121908699	1 (0.3)	0	-	-
i3	c.592+50A>T	rs17881298	0	2 (0.5)	-	-
i4	c.684-78_-100dup23	rs17881244	23 (6.8)	27 (6.1)	1.12 (0.63–2.00)	0.77
E8	c.990G>A (p.Q330Q)	rs9625537	0	1 (0.2)	-	-
i8	c.1008+71A>G ^a^	rs121908713	1 (0.3)	0	-	-
i9	c.1095+19G>A	rs200020484	0	2 (0.5)	-	-
i9	c.1095+99A>C	-	0	1 (0.2)	-	-
i9	c.1096-18T>C	-	0	1 (0.2)	-	-
i10	c.1259+28A>G ^a^	rs121908708	1 (0.3)	0	-	-
i11	c.1375+16G>A	-	0	1 (0.2)	-	-
i13	c.1543-31delT ^a^	rs121908709	1 (0.3)	0	-	-
3’UTR	c.*7T>C ^a^	rs121908710	1 (0.3)	0	-	-

^a^ New alterations

^b^The c.319+43dupA alteration also did not show a statistically significant deviation from the Hardy-Weinberg equilibrium in any of the analyzed groups (all p > 0.05).

Because germline alterations affecting CHK2 protein sequence were found more frequently in NHL patients, they were associated with an increased risk of NHL development (OR = 2.86; 95% CI 1.42–5.79; Table 2). These alterations were scattered among various types of NHL; as a result, despite relatively small numbers of patients for each type, their association with NHL was significant for follicular lymphoma (FL; *P* = 0.03), mantle cell lymphoma (MCL; *P* = 0.02), and B-small lymphocytic lymphoma (B-SLL; *P* = 0.004), and borderline for diffuse large B-cell lymphoma (DLBCL; *P* = 0.06; S3 Table). After excluding the most frequent missense variant I157T, the remaining relatively rare non-I157T CHK2 protein-modifying alterations were still significantly associated with an increased NHL risk (OR = 8.10; 95% CI 1.80–36.47; *P =* 0.002).

Interestingly, the carriers of at least one allele of the most frequent intronic variant c.319+43dupA were at a significantly lower risk of NHL development (OR = 0.62; 95% CI 0.45–0.86, *P* = 0.005). The protective effect of c.319+43dupA was also of borderline statistical significance in the two major types of NHL: DLBCL and FL (both *P =* 0.05; S3 Table).

### Correlation of *CHEK2* alterations with survival of NHL patients

**Fig 2:**
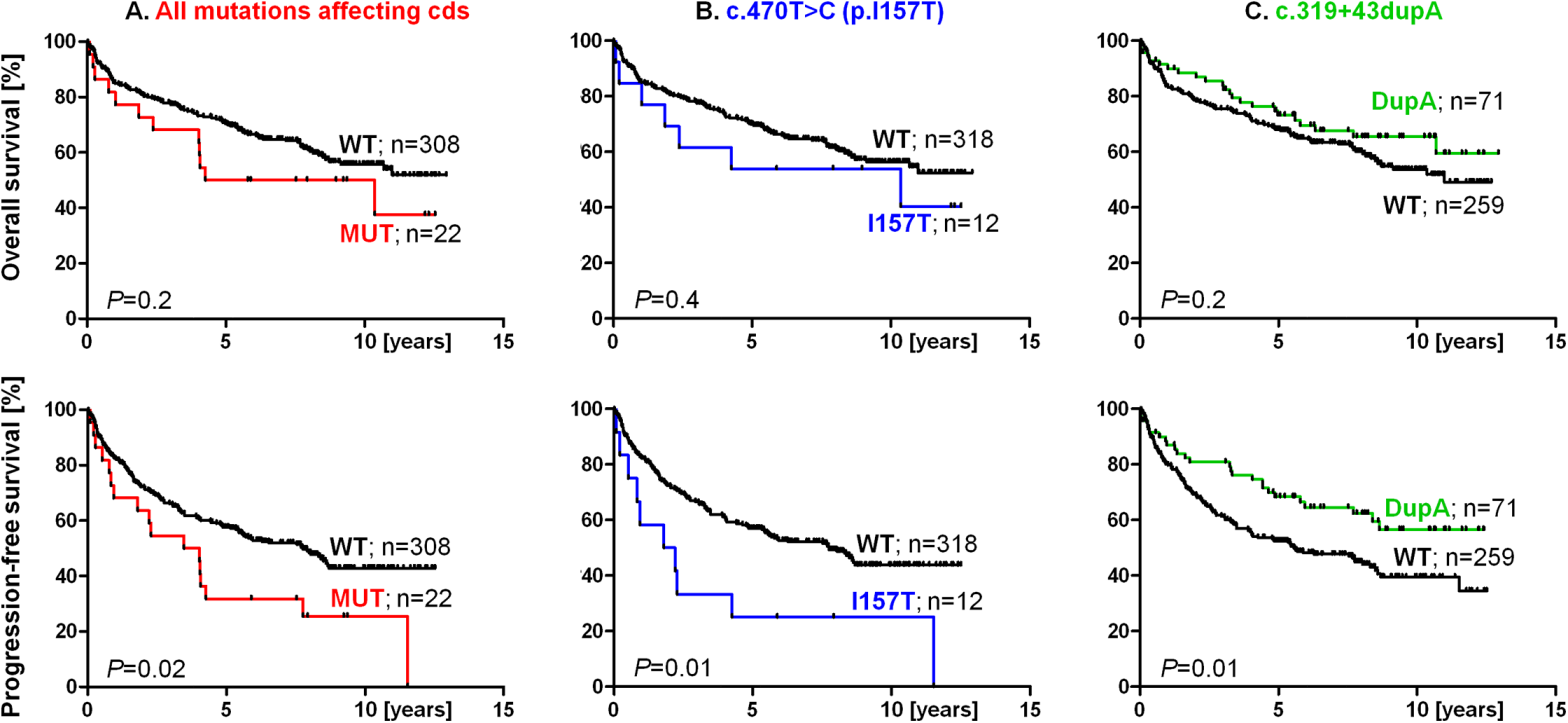
Overall survival (OS; upper panels) and progression-free survival (PFS; lower panels) in all NHL patients (regardless of histology subtype) classified according to the type of *CHEK2* alterations. Panels show: **A.** the influence of all alterations affecting the CHK2 coding sequence (cds; HR_OS_ = 1.6; 95% CI 0.79–3.24 and HR_PFS_ = 2.1; 95% CI 1.12–4.05); **B.** the influence of the I157T mutation (HR_OS_ = 1.5; 95% CI 0.62–3.70 and HR_PFS_ = 3.7; 95% CI 1.42–9.43) and **C.** the influence of the c.319+43dupA variant (HR_OS_ = 0.8; 95% CI 0.50–1.15 and HR_PFS_ = 0.6; 95% CI 0.44–0.89).

All identified *CHEK2* alterations were evaluated as potential prognostic factors modifying the survival of NHL patients. We found that CHK2 protein-modifying alterations were associated with worse progression-free survival (PFS) of NHL patients overall (i.e., regardless of histology type; HR_PFS_ = 2.1; P = 0.02; Fig 2A), and that the association was even stronger for carriers of the missense variant I157T compared with any other genotype (i.e. I157T vs. mutation-free or any other alteration; HR_PFS_ = 3.7 *P* = 0.007; Fig 2B). Similar but statistically insignificant results were observed for overall survival (OS, Fig 2A and 2B).

**Fig 3:**
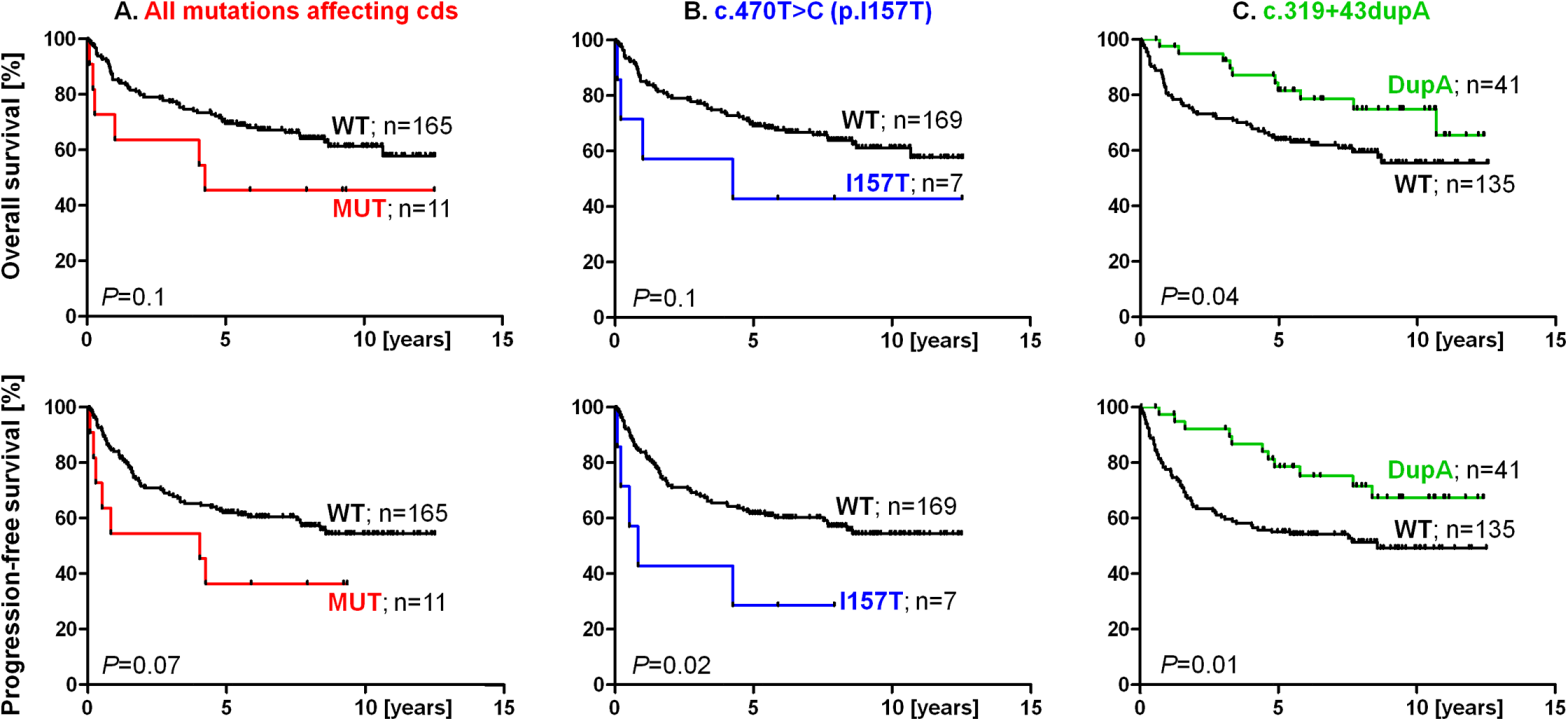
Overall survival (OS; upper panels) and progression-free survival (PFS; lower panels) in DLBCL patients. Panels show: **A.** the influence of all alterations affecting the CHK2 coding sequence (cds; HR_OS_ = 2.3; 95% CI 0.77–6.97 and HR_PFS_ = 2.6; 95% CI 0.91–7.44); **B.** the influence of the I157T mutation (HR_OS_ = 2.9; 95% CI 0.70–12.00 and HR_PFS_ = 5.2; 95% CI 1.25–22.16) and **C.** the influence of the c.319+43dupA variant (HR_OS_ = 0.6; 95% CI 0.32–0.97 and HR_PFS_ = 0.5; 95% CI 0.32–0.86).

We subsequently focused on 180 DLBCL patients, representing the most common type of NHL. The I157T mutation negatively affected PFS also in the DLBCL subgroup (HR_PFS_ = 5.2; *P* = 0.02; Fig 3B), but not significantly the OS. There was no statistically significant difference in survival between DLBCL patients with or without any germline mutation affecting CHK2 protein sequence (Fig 3A). The I157T mutation was also associated with a higher number of lymph node areas affected by the tumor in DLBCL patients (risk of more than four areas OR = 9.7; 95% CI 1.8–52.2; *P =* 0.008).

In contrast to CHK2 protein-modifying mutations, the most frequent *CHEK2* alteration c.319+43dupA (carried by 22% of NHL patients and associated with a decreased risk of NHL development) was significantly associated with better OS and PFS in DLBCL patients: HR_OS_ = 0.6 (*P* = 0.04) and HR_PFS_ = 0.5 (*P* = 0.01; Fig 3C). The association of c.319+43dupA with better PFS was also significant for the entire group of NHL patients, but c.319+43dupA did not modify OS in this group (Fig 2C). The c.319+43dupA alteration did not modify OS or PFS in any other NHL types (all *P* values were higher than 0.05) or in all NHL patients excluding DLBCL (HR_OS_ = 1.85; *P* = 0.1; HR_PFS_ = 0.97; *P* = 0.6). Therefore, its impact was limited to the DLBCL subgroup only. The c.319+43dupA alteration was also an independent positive prognostic factor for the DLBCL OS even when the International Prognostic Index (IPI) was included in the analysis: HR_OS,DupA_ = 0.38 (95% CI 0.16–0.94; *P* = 0.04) and high/intermediate high IPI HR_OS_ = 3.57 (95% CI 2.03–6.28; *P*<0.0001). DLBCL carriers of the c.319+43dupA tended to be younger at NHL diagnosis: the median age at diagnosis of c.319+43dupA carriers (heterozygotes + homozygotes together) vs. all NHL patients without this alteration was 54.5 vs. 59.6 years, respectively (*P* = 0.02). The carriers of at least one allele with the c.319+43dupA alteration had also higher probability of bone marrow negativity (OR = 2.1; 95% CI 1.2–3.9; *P* = 0.02). Both these clinical factors could contribute to better survival of DLBCL patients. The type of therapy was essentially identical between carriers and non-carriers of c.319+43dupA. The majority of DLBCL patients were treated by chemotherapy classifiable as CHOP (cyclophosphamide, doxorubicin, vincristine, and prednisolone; S4 Table), but the protective effect of c.319+43dupA was the strongest and limited to patients treated by conventional chemotherapy only (HR_OS_ = 0.4; P = 0.03; HR_PFS_ = 0.4; P = 0.006), while the presence of c.319+43dupA did not modify survival in the DLBCL subgroup treated by rituximab-based chemoimmunotherapy (S4A and S4B Fig). Analysis of the additional 186 DLBCL patients (validation DLBCL group, Table 1), where 95% patients were treated by rituximab (S4 Table), confirmed that c.319+43dupA do not affect survival of rituximab treated patients (S4C Fig). Analysis of CHEK2 mRNA (resp. cDNA) from the carriers of c.319+43dupA (homozygotes or heterozygotes) did not show any aberration of CHEK2 pre-mRNA slicing (data not shown); hence, the biological importance of this variant remains elusive.

We did not find any other correlation of a particular *CHEK2* alteration or group of alterations with other evaluated clinical characteristics (age, gender, clinical stage, IPI, FLIPI, bone marrow involvement, elevated LDH, number of lymph nodes areas affected by the tumor, extranodal involvement, maximum tumor diameter, and best response to treatment).

## Discussion

Non-Hodgkin lymphomas occur only rarely in an apparently familial form (OMIM 605027), and therefore it is unlikely that a strong, clinically meaningful high-penetrant NHL-susceptibility gene will be found. On the other hand, the influence of various low and/or medium-penetrant alleles on the risk of NHL has been documented [6]. A recent report describes germline polymorphisms in the LUBAC subunit RNF31, rare among healthy individuals (∼1%) but enriched in the ABC subtype of DLBCL (7.8%), that were shown by in vitro studies as likely contributory to the disease and providing a therapeutic target [29].

Germline alterations of the *CHEK2* gene were associated with a higher risk of several solid tumor types, but their association with hematological malignances was inconsistent or limited [7, 30]. We analyzed hereditary *CHEK2* variants in the entire coding sequence of the *CHEK2* gene in large group of NHL patients, and found that *CHEK2* variants could modify the risk and clinical course of NHL. Specifically, 7.4% of NHL patients are carriers of alterations affecting the CHK2 protein sequence, which are associated with an increased risk of NHL development (OR = 2.86; *P =* 0.003). Interestingly, this association was apparent also for major individual types of NHL, suggesting that the effect of CHK2 protein-modifying alterations is not limited to a particular type of NHL. Five out of 340 NHL patients (1.5%) carried a variant truncating the CHK2 protein sequence, in all cases upstream of the kinase domain, similarly to the 1100delC alteration (a well-known pathogenic mutation increasing the risk of various solid cancers). Therefore, a follow-up with specialized oncologists and genetic counseling for the families of these patients is highly recommended [31]. Two cases of large deletion (5395 del mutation), previously described as Slavic population-specific variant for its origin from Czech or Slovak regions [21], were found by a MLPA. Our results indicate that with the exception of 5395 del, large genomic rearrangements in the *CHEK2* gene are rare events. Other than this intragenic deletion, a 23 Kb duplication in a single Italian breast cancer patient is the only other known *CHEK2* rearrangement variant [32]. The presence of 5395 del in our NHL patients also indicates that this variant may not be associated only with breast [33] or prostate [34] cancer susceptibility. After the exclusion of clearly pathogenic truncating variants and the most frequent missense mutation I157T (whose frequency was not significantly different between our NHL patients and controls), all other relatively rare *CHEK2* missense variants occurred more frequently in the patients’ group (Fig 1) and were also associated with an increased NHL risk (OR = 4.66; *P =* 0.045). A similar effect of such rare *CHEK2* variants has been reported for colorectal [22] and breast cancer [18, 35], supporting the importance of less frequent alterations in not-commonly analyzed *CHEK2* regions.

Our results are in accordance with the study by Cybulski, *et al* (2005), who analyzed tree founder *CHEK2* variants, p.I157T, IVS2+1G>A (c.444+1G>A) and c.1100delC, in a large population of cancer patients with various tumor types that included also a subgroup of 120 NHL cases [7]. In this subset, the I157T variant showed a borderline association with an increased risk of NHL development (OR 2.0; *P =* 0.05). In our study, the frequency of this variant was also higher in NHL cases compared with controls, but the difference was not statistically significant (4.1% vs. 2.2%, respectively; *P* = 0.15). The overall frequency of I157T was, however, considerably higher in the Polish compared with the Czech population (9.2% vs. 4.8%, respectively). Besides our work and the Cybulski report [7], all other studies on NHL patients analyzed primarily somatic *CHEK2* mutations [36–38]. A study by [39] is of particular interest because it describes *CHEK2* and other DNA repair genes being selectively somatically mutated in DLBCL tumors and thus supports the importance of the DDR system in NHL development. From a wider perspective, NHL might not be the only hematological malignancy associated with *CHEK2* germline alterations. We have recently reported an association of alterations localized within the *CHEK2* FHA domain coding region with an increased risk of Hodgkin lymphoma (OR = 2.11; *P =* 0.04; [40]. Further, *CHEK2* mutations (together with other genes implicated in DNA repair) were described as part of mutational landscape of T-cell prolymphocytic leukemia [41]. Janiszewska et al. suggested the contribution of four selected germline mutations (I157T, 1100delC, IVS2+1G>A (c.444+1G>A) and del5395) to the development of essential thrombocythemia (OR = 3.8; *P =* 0.002) [42]. Recently, they also identified an increased risk of polycythaemia vera (OR = 3.0; *P* = 0.004) in carriers of aforementioned *CHEK2* variants in Polish population [43]. A strong association was also reported for the *CHEK2* I157T mutation and an increased risk of chronic lymphocytic leukemia (OR = 14.83; *P =* 0.0008) [30].

Beyond NHL risk, alterations in the *CHEK2* gene have not been evaluated as a factor modifying NHL survival previously. We found germline mutations altering CHK2 protein sequence to be associated not only with an increased risk of NHL development, but also with unfavorable NHL survival in mutation carriers (NHL HR_PFS_ = 2.1; *P* = 0.02, Fig 2A). A similar pattern was also observed for the most frequent missense variant I157T alone and for CHK2 protein-modifying alterations in the most common NHL type, DLBCL (Figs 2B, 3A and 3B). *CHEK2* mutations have previously been linked to chemotherapy resistance: Chrisanthar et al. [44] reported an association of *CHEK2* mutations with resistance to anthracycline (doxorubicin and epirubicin) therapy in primary breast cancer and shown that decreased downstream p53 activation may contribute to anthracycline resistance in cancers with somatic deletion of wt *CHEK2* allele (loss of heterozygosity; LOH). A similar effect might be responsible for inferior PFS in NHL, for which doxorubicin is an essential part of most chemotherapy regimens; however, this hypothesis needs to be further investigated by LOH analyses in NHL tumor specimens of *CHEK2* hereditary mutation carriers (not available in our study) and especially by *in vivo* models. Changes in CHK2 function could also affect the efficacy of other agents used for NHL treatment (e.g., cyclophosphamide), since CHK2 is a member of a pathway executing response to DNA-damaging agents in general [45].

The c.319+43dupA alteration was the only variant significantly associated with a decreased risk of NHL (OR = 0.62; *P* = 0.005). Interestingly, our analysis suggested that c.319+43dupA might be also an independent positive prognostic factor associated with better OS and PFS of DLBCL, but the protective effect of c.319+43dupA on survival was limited to patients treated by conventional chemotherapy only (without rituximab; S4 Fig). It indicates that adding rituximab to the therapeutic regimen might overcome chemotherapy-resistance highlighted by the absence of c.319+43dupA alteration, and that the carriers of this alteration may respond to conventional chemotherapy well even without rituximab. Similar effect of rituximab was reported for negative prognostic impact of cyclin E expression in DLBCL [46]. Longer follow-up and/or a larger group of patients might be needed to detect eventual differences in survival associated with the c.319+43dupA polymorphism in the era of rituximab that substantially improved the overall survival rates. The mechanistic and biological rationale of how the *CHEK2* c.319+43dupA allele may modify the CHK2 function is unclear. Computer predictions and analysis of mRNA did not reveal interaction of this variant with *CHEK2* pre-mRNA splicing (data not shown). However, other processes influencing gene transcription (at the level of intronic splicing enhancers/silencers) or mRNA metabolism (RNA interference, stability or processing) may be involved. Moreover, the effect of another locus in linkage with c.319+43dupA cannot be ruled out.

In conclusion, our results indicate that relatively frequent germline *CHEK2* mutations (present in 7.4% of NHL patients) are associated with a moderately increased risk of NHL and unfavorable prognosis. However, further independent studies in NHL patients are required to confirm this association and the biological consequences of *CHEK2* mutations in the development of NHL. We have also shown that germline mutations of the *CHEK2* gene are not restricted to the previously analyzed regions (FHA and kinase domains) but are scattered across the entire coding sequence, and that intragenic rearrangements in the *CHEK2* gene are rare events with only two Slavic founder mutations (5395 del) and no other types detected in our analysis. The c.319+43dupA polymorphism was associated with a decreased risk of NHL development; however, the role of c.319+43dupA as an independent marker of positive prognosis in DLBCL needs further investigations.

## Supporting Information

S1 Fig Identification of the c.444+1G>T variant. The *CHEK2* mutation c.444+1G>T (MUT) as it appeared in a DHPLC analysis (A) performed at 56.5°C. Sequencing chromatograms showing the sequence of this variant amplified form gDNA (B) and RNA (resp. cDNA; C). G-to-T transversion affecting the first nucleotide in the intron 2 (low letters) results in a selection of the aberrant splicing “gt” dinucleotide (underlined in B) that results in the formation of mutant mRNA containing a deleted last guanosine from exon 2 (depicted in C) which leads to the frame-shift and premature termination of translation (p.E149Kfs*12). The lower signal of the mutant c.444+1G>T allele (C) compared with the wild type (WT) allele signal in amplicons from cDNA synthesized from the peripheral blood leucocytes’ total RNA of the c.444+1G>T mutation carrier may be the result of the nonsense-mediated decay activity depredating mutation-bearing mRNA that contains the premature termination codon.
(TIF)

S2 Fig Identification of the c.1259+1G>C variant. The *CHEK2* mutation c.1259+1G>C (MUT) as it appeared in DHPLC analysis (A) performed at 56.2°C. Sequencing chromatograms showing the sequence of this variant amplified form gDNA (B) and RNA (resp. cDNA; C, showing the borders of aberrant splicing product sequenced with forward (left) and reverse (right) sequencing primers). G-to-C transversion affecting the first nucleotide in the intron 10 (low letters) results in the cessation of the intron 10 donor splice site. This causes aberrant splicing with exon 10 skipping (C) that results in the frame-shift and premature termination of translation (p.I336Pfs*2).
(TIF)

S3 Fig Identification of LGR using MLPA. Results of the MLPA analysis using the Coffalyser software showing a sample with a wild-type *CHEK2* sequence (A) and a sample carrying a large 5395 bp deletion (B) affecting the exons 8 and 9 (denominated in MLPA as exons 9 and 10, red bars).
(TIF)

S4 Fig Kaplan-Meier estimates of overall survival (OS; upper panels) and progression-free survival (PFS, lower panels) in patients from the originally tested DLBCL subgroup (A and B) and the DLBCL validation group (C) classified according to the presence of the c.319+43dupA variant. Panels show the OS and PFS in: A. patients treated only by conventional chemotherapy (HR_OS_ = 0.4; 95% CI 0.18–0.91; HR_PFS_ = 0.4; 95% CI 0.17–0.74), B. patients treated by rituximab-based chemoimmunotherapy (RTX; HR_OS_ = 0.8; 95% CI 0.36–1.17; HR_PFS_ = 0.7; 95% CI 0.36–1.50), and C. all patients from the DLBCL validation group (95% of patients treated with rituximab-based regimen; HR_OS_ = 0.8; 95% CI 0.40–1.39; HR_PFS_ = 0.8; 95% CI 0.47–1.40).
(TIF)

S1 Table Conditions for PCR amplification of *CHEK2* coding sequence and following DHPLC analysis. (PDF)

S2 Table Predicted effects of the *CHEK2* missense variants identified in NHL patients in this study using software prediction. (PDF)

S3 Table Risk of NHL subtypes development in carriers of mutations modifying CHK2 protein structure and c.319+43dupA variant. (PDF)

S4 Table Comparison of therapy type between c.319+43dupA carriers and non-carriers (original group of DLBCL patients) and between two analyzed DLBCL groups. (PDF)

S5 Table Results of mutational analysis in 340 NHL patients showing individual CHEK2 genotypes. (XLSX)

## References

[1] Parkin DM, Bray F, Ferlay J, Pisani P (2005) Global cancer statistics, 2002. CA Cancer J Clin 55:74–108. 15761078 10.3322/canjclin.55.2.74

[2] Dusek L, Muzik J, Gelnarova E, Finek J, Vyzula R, Abrahamova J (2010) Cancer incidence and mortality in the Czech Republic. Klin Onkol 23:311–324. 21058527

[3] Kadan-Lottick NS, Kawashima T, Tomlinson G, Friedman DL, Yasui Y, Mertens AC, et al (2006) The risk of cancer in twins: a report from the childhood cancer survivor study. Pediatr Blood Cancer 46:476–481. 16078231 10.1002/pbc.20465

[4] Zhang Y, Wang R, Holford TR, Leaderer B, Zahm SH, Boyle P, et al (2007) Family history of hematopoietic and non-hematopoietic malignancies and risk of non-Hodgkin lymphoma. Cancer Causes Control 18:351–359. 17206533 10.1007/s10552-006-0088-5

[5] Wang SS, Slager SL, Brennan P, Holly EA, De Sanjose S, Bernstein L, et al (2007) Family history of hematopoietic malignancies and risk of non-Hodgkin lymphoma (NHL): a pooled analysis of 10 211 cases and 11 905 controls from the International Lymphoma Epidemiology Consortium (InterLymph). Blood 109:3479–3488. 17185468 10.1182/blood-2006-06-031948 PMC1852242

[6] Skibola CF, Curry JD, Nieters A (2007) Genetic susceptibility to lymphoma. Haematologica 92:960–9. 17606447 10.3324/haematol.11011 PMC2819165

[7] Cybulski C, Gorski B, Huzarski T, Masojc B, Mierzejewski M, Debniak T, et al (2004) CHEK2 is a multiorgan cancer susceptibility gene. Am J Hum Genet 75:1131–1135. 15492928 10.1086/426403 PMC1182149

[8] Cai Z, Chehab NH, Pavletich NP (2009) Structure and activation mechanism of the CHK2 DNA damage checkpoint kinase. Mol Cell 35:818–829. 10.1016/j.molcel.2009.09.007 19782031

[9] Prokopcova J, Kleibl Z, Banwell CM, Pohlreich P (2007) The role of ATM in breast cancer development. Breast Cancer Res Treat 104:121–128. 17061036 10.1007/s10549-006-9406-6

[10] Bartkova J, Horejsi Z, Koed K, Kramer A, Tort F, Zieger K, et al (2005) DNA damage response as a candidate anti-cancer barrier in early human tumorigenesis. Nature 434:864–870. 15829956 10.1038/nature03482

[11] Gorgoulis VG, Vassiliou LV, Karakaidos P, Zacharatos P, Kotsinas A, Liloglou T, et al (2005) Activation of the DNA damage checkpoint and genomic instability in human precancerous lesions. Nature 434:907–913. 15829965 10.1038/nature03485

[12] Bartek J, Bartkova J, Lukas J (2007) DNA damage signalling guards against activated oncogenes and tumour progression. Oncogene 26:7773–7779. 18066090 10.1038/sj.onc.1210881

[13] Hanahan D, Weinberg RA (2011) Hallmarks of cancer: the next generation. Cell 144:646–674. 10.1016/j.cell.2011.02.013 21376230

[14] Ham MF, Takakuwa T, Luo WJ, Liu A, Horii A, Aozasa K (2006) Impairment of double-strand breaks repair and aberrant splicing of ATM and MRE11 in leukemia-lymphoma cell lines with microsatellite instability. Cancer Sci 97:226–234. 16542220 10.1111/j.1349-7006.2006.00165.x PMC11159514

[15] Bendall LJ (2011) It's all in the timing. Blood 118:5983–5984. 10.1182/blood-2011-09-381111 22134906

[16] Taylor AM, Metcalfe JA, Thick J, Mak YF (1996) Leukemia and lymphoma in ataxia telangiectasia. Blood 87:423–438. 8555463

[17] Gumy-Pause F, Wacker P, Maillet P, Betts DR, Sappino AP (2006) ATM alterations in childhood non-Hodgkin lymphoma. Cancer Genet Cytogenet 166:101–111. 16631465 10.1016/j.cancergencyto.2005.09.005

[18] Kleibl Z, Havranek O, Novotny J, Kleiblova P, Soucek P, Pohlreich P (2008) Analysis of CHEK2 FHA domain in Czech patients with sporadic breast cancer revealed distinct rare genetic alterations. Breast Cancer Res Treat 112:159–164. 18058223 10.1007/s10549-007-9838-7

[19] Sodha N, Houlston RS, Williams R, Yuille MA, Mangion J, Eeles RA (2002) A robust method for detecting CHK2/RAD53 mutations in genomic DNA. HumMutat 19:173–177. 10.1002/humu.10031 11793476

[20] Sodha N, Williams R, Mangion J, Bullock SL, Yuille MR, Eeles RA, et al (2000) Screening hCHK2 for Mutations. Science 289:359 10939935 10.1126/science.289.5478.359a

[21] Walsh T, Casadei S, Coats KH, Swisher E, Stray SM, Higgins J, et al (2006) Spectrum of mutations in BRCA1, BRCA2, CHEK2, and TP53 in families at high risk of breast cancer. JAMA 295:1379–1388. 16551709 10.1001/jama.295.12.1379

[22] Kleibl Z, Havranek O, Hlavata I, Novotny J, Sevcik J, Pohlreich P, et al (2009) The CHEK2 gene I157T mutation and other alterations in its proximity increase the risk of sporadic colorectal cancer in the Czech population. Eur J Cancer 45:618–624. 10.1016/j.ejca.2008.09.022 18996005

[23] Kleiblova P, Dostalova I, Bartlova M, Lacinova Z, Ticha I, Krejci V, et al (2010) Expression of adipokines and estrogen receptors in adipose tissue and placenta of patients with gestational diabetes mellitus. Mol Cell Endocrinol 314:150–156. 10.1016/j.mce.2009.08.002 19682537

[24] Tavtigian SV, Byrnes GB, Goldgar DE, Thomas A (2008) Classification of rare missense substitutions, using risk surfaces, with genetic- and molecular-epidemiology applications. Hum Mutat 29:1342–1354. 10.1002/humu.20896 18951461 PMC3938023

[25] Kumar P, Henikoff S, Ng PC (2009) Predicting the effects of coding non-synonymous variants on protein function using the SIFT algorithm. Nat Protoc 4:1073–1081. 10.1038/nprot.2009.86 19561590

[26] Adzhubei IA, Schmidt S, Peshkin L, Ramensky VE, Gerasimova A, Bork P, et al (2010) A method and server for predicting damaging missense mutations. Nat Methods 7:248–249. 10.1038/nmeth0410-248 20354512 PMC2855889

[27] Schwarz JM, Rodelsperger C, Schuelke M, Seelow D. (2010) MutationTaster evaluates disease-causing potential of sequence alterations. Nat Methods 7:575–576. 10.1038/nmeth0810-575 20676075

[28] Houdayer C (2011) In silico prediction of splice-affecting nucleotide variants. Methods Mol Biol 760:269–281. 10.1007/978-1-61779-176-5_17 21780003

[29] Yang Y, Schmitz R, Mitala J, Whiting A, Xiao W, Ceribelli M, et al (2014) Essential role of the linear ubiquitin chain assembly complex in lymphoma revealed by rare germline polymorphisms. Cancer Discov 4:480–493. 10.1158/2159-8290.CD-13-0915 24491438 PMC3992927

[30] Rudd MF, Sellick GS, Webb EL, Catovsky D, Houlston RS (2006) Variants in the ATM-BRCA2-CHEK2 axis predispose to chronic lymphocytic leukemia. Blood 108:638–644. 16574953 10.1182/blood-2005-12-5022

[31] Narod SA (2010) Testing for CHEK2 in the cancer genetics clinic: ready for prime time? Clin Genet 78:1–7. 10.1111/j.1399-0004.2010.01402.x 20597917

[32] Tedaldi G, Danesi R, Zampiga V, Tebaldi M, Bedei L, Zoli W, et al (2014) First evidence of a large CHEK2 duplication involved in cancer predisposition in an Italian family with hereditary breast cancer. BMC Cancer 14:478 10.1186/1471-2407-14-478 24986639 PMC4091954

[33] Cybulski C, Wokolorczyk D, Huzarski T, Byrski T, Gronwald J, Gorski B, et al (2006) A deletion in CHEK2 of 5,395 bp predisposes to breast cancer in Poland. Breast Cancer Res Treat 102:119–122. 16897426 10.1007/s10549-006-9320-y

[34] Cybulski C, Wokolorczyk D, Huzarski T, Byrski T, Gronwald J, Gorski B, et al (2006) A large germline deletion in the Chek2 kinase gene is associated with an increased risk of prostate cancer. J Med Genet 43:863–866. 17085682 10.1136/jmg.2006.044974 PMC2563179

[35] Le Calvez-Kelm F, Lesueur F, Damiola F, Vallee M, Voegele C, Babikyan D, et al (2011) Rare, evolutionarily unlikely missense substitutions in CHEK2 contribute to breast cancer susceptibility: results from a breast cancer family registry case-control mutation-screening study. Breast Cancer Res 13:R6 10.1186/bcr2810 21244692 PMC3109572

[36] Hangaishi A, Ogawa S, Qiao Y, Wang L, Hosoya N, Yuji K, et al (2002) Mutations of Chk2 in primary hematopoietic neoplasms. Blood 99:3075–3077. 11949635 10.1182/blood.v99.8.3075

[37] Tavor S, Takeuchi S, Tsukasaki K, Miller CW, Hofmann WK, Ikezoe T, et al (2001) Analysis of the CHK2 gene in lymphoid malignancies. Leuk Lymphoma 42:517–520. 11699418 10.3109/10428190109064610

[38] Tort F, Hernandez S, Bea S, Martinez A, Esteller M, Herman JG, et al (2002) CHK2-decreased protein expression and infrequent genetic alterations mainly occur in aggressive types of non-Hodgkin lymphomas. Blood 100:4602–4608. 12393693 10.1182/blood-2002-04-1078

[39] de Miranda NF, Peng R, Georgiou K, Wu C, Falk Sorqvist E, Berglund M, et al (2013) DNA repair genes are selectively mutated in diffuse large B cell lymphomas. J Exp Med 210:1729–1742. 10.1084/jem.20122842 23960188 PMC3754869

[40] Havranek O, Spacek M, Hubacek P, Mocikova H, Markova J, Trneny M, et al (2011) Alterations of CHEK2 forkhead-associated domain increase the risk of Hodgkin lymphoma. Neoplasma. 58:392–395. 21744992 10.4149/neo_2011_05_392

[41] Kiel MJ, Velusamy T, Rolland D, Sahasrabuddhe AA, Chung F, Bailey NG, et al (2014) Integrated genomic sequencing reveals mutational landscape of T-cell prolymphocytic leukemia. Blood 124:1460–1472. 10.1182/blood-2014-03-559542 24825865 PMC4148768

[42] Janiszewska H, Bak A, Pilarska M, Heise M, Junkiert-Czarnecka A, Kuliszkiewicz-Janus M, et al (2012) A risk of essential thrombocythemia in carriers of constitutional CHEK2 gene mutations. Haematologica 97:366–370. 10.3324/haematol.2011.049494 22058216 PMC3291590

[43] Janiszewska H, Bąk A, Hartwig M, Kuliszkiewicz-Janus M, Całbecka M, Jaźwiec B, et al The germline mutations of theCHEK2gene are associated with an increased risk of polycythaemia vera. Br J Haematol: 10.1111/bjh.13559 26084796

[44] Chrisanthar R, Knappskog S, Lokkevik E, Anker G, Ostenstad B, Lundgren S, et al (2008) CHEK2 mutations affecting kinase activity together with mutations in TP53 indicate a functional pathway associated with resistance to epirubicin in primary breast cancer. PLoS One 3:e3062 10.1371/journal.pone.0003062 18725978 PMC2518116

[45] Antoni L, Sodha N, Collins I, Garrett MD (2007) CHK2 kinase: cancer susceptibility and cancer therapy—two sides of the same coin? Nat Rev Cancer 7:925–936. 18004398 10.1038/nrc2251

[46] Frei E, Visco C, Xu-Monette ZY, Dirnhofer S, Dybkaer K, Orazi A, et al (2013) Addition of rituximab to chemotherapy overcomes the negative prognostic impact of cyclin E expression in diffuse large B-cell lymphoma. J Clin Pathol 66:956–961. 10.1136/jclinpath-2013-201619 23775435

