# Supplementary figures and images for: Association of Germline CHEK2 Gene Variants with Risk and Prognosis of Non-Hodgkin Lymphoma
Source: PLoS One. 2015 Oct 27;10(10):e0140819. doi: 10.1371/journal.pone.0140819 (PMC4624763; doi:10.1371/journal.pone.0140819)

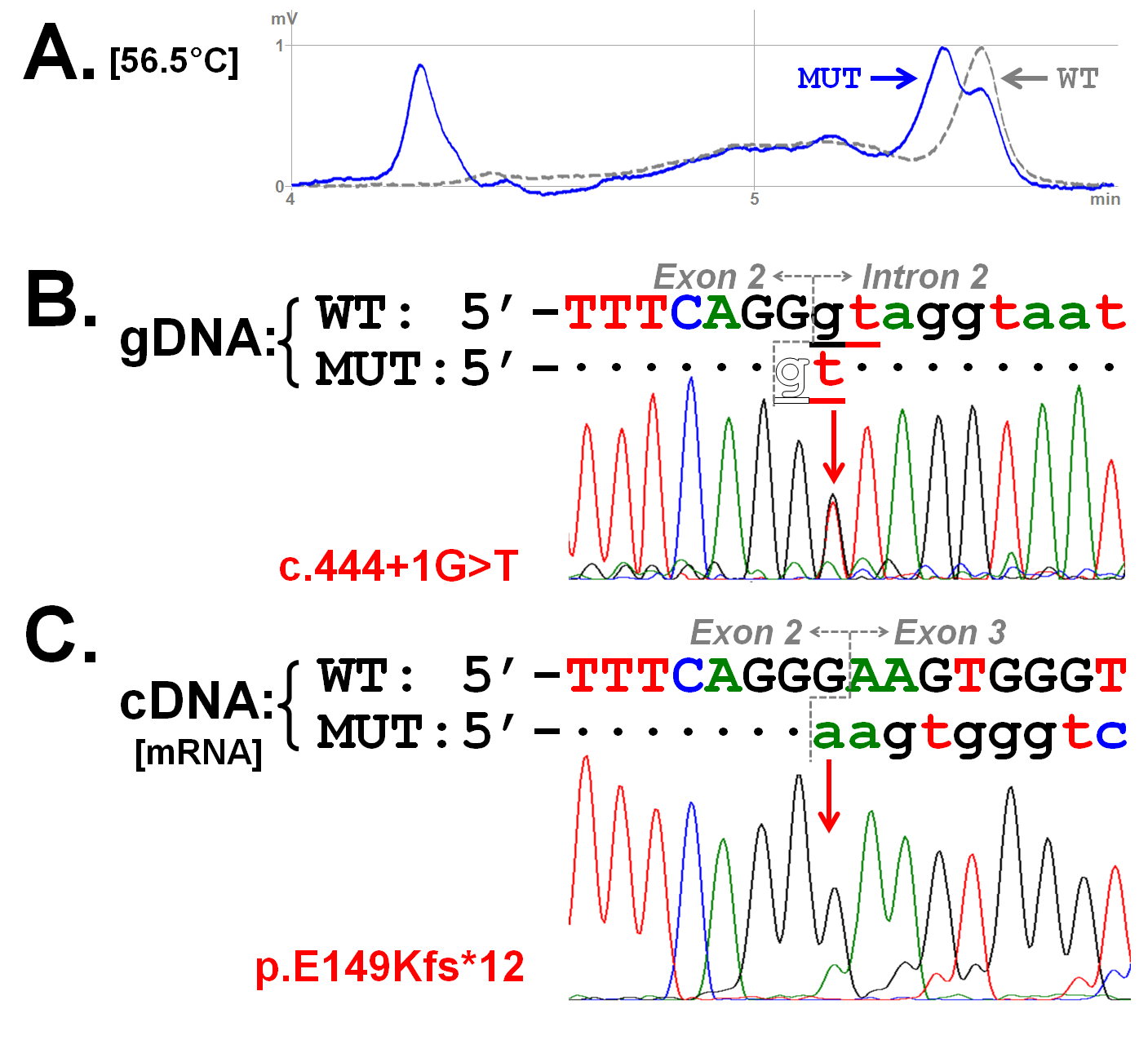

Supplement: S1 Fig — The CHEK2 mutation c.444+1G>T (MUT) as it appeared in a DHPLC analysis (A) performed at 56.5°C. Sequencing chromatograms showing the sequence of this variant amplified form gDNA (B) and RNA (resp. cDNA; C). G-to-T transversion affecting the first nucleotide in the intron 2 (low letters) results in a selection of the aberrant splicing “gt” dinucleotide (underlined in B) that results in the formation of mutant mRNA containing a deleted last guanosine from exon 2 (depicted in C) which leads to the frame-shift and premature termination of translation (p.E149Kfs*12). The lower signal of the mutant c.444+1G>T allele (C) compared with the wild type (WT) allele signal in amplicons from cDNA synthesized from the peripheral blood leucocytes’ total RNA of the c.444+1G>T mutation carrier may be the result of the nonsense-mediated decay activity depredating mutation-bearing mRNA that contains the premature termination codon. (TIF) [file pone.0140819.s001.tif]

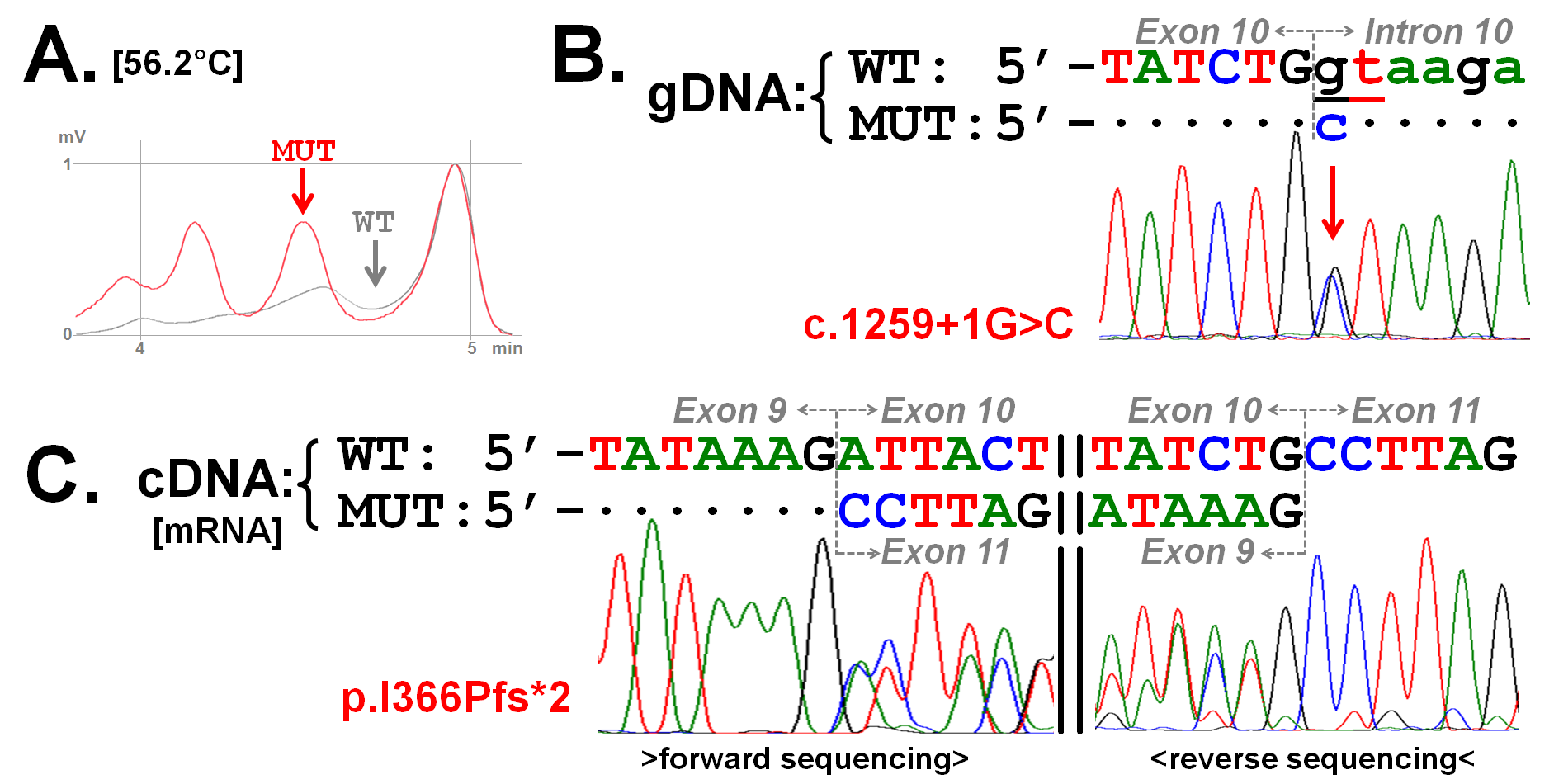

Supplement: S2 Fig — The CHEK2 mutation c.1259+1G>C (MUT) as it appeared in DHPLC analysis (A) performed at 56.2°C. Sequencing chromatograms showing the sequence of this variant amplified form gDNA (B) and RNA (resp. cDNA; C, showing the borders of aberrant splicing product sequenced with forward (left) and reverse (right) sequencing primers). G-to-C transversion affecting the first nucleotide in the intron 10 (low letters) results in the cessation of the intron 10 donor splice site. This causes aberrant splicing with exon 10 skipping (C) that results in the frame-shift and premature termination of translation (p.I336Pfs*2). (TIF) [file pone.0140819.s002.tif]

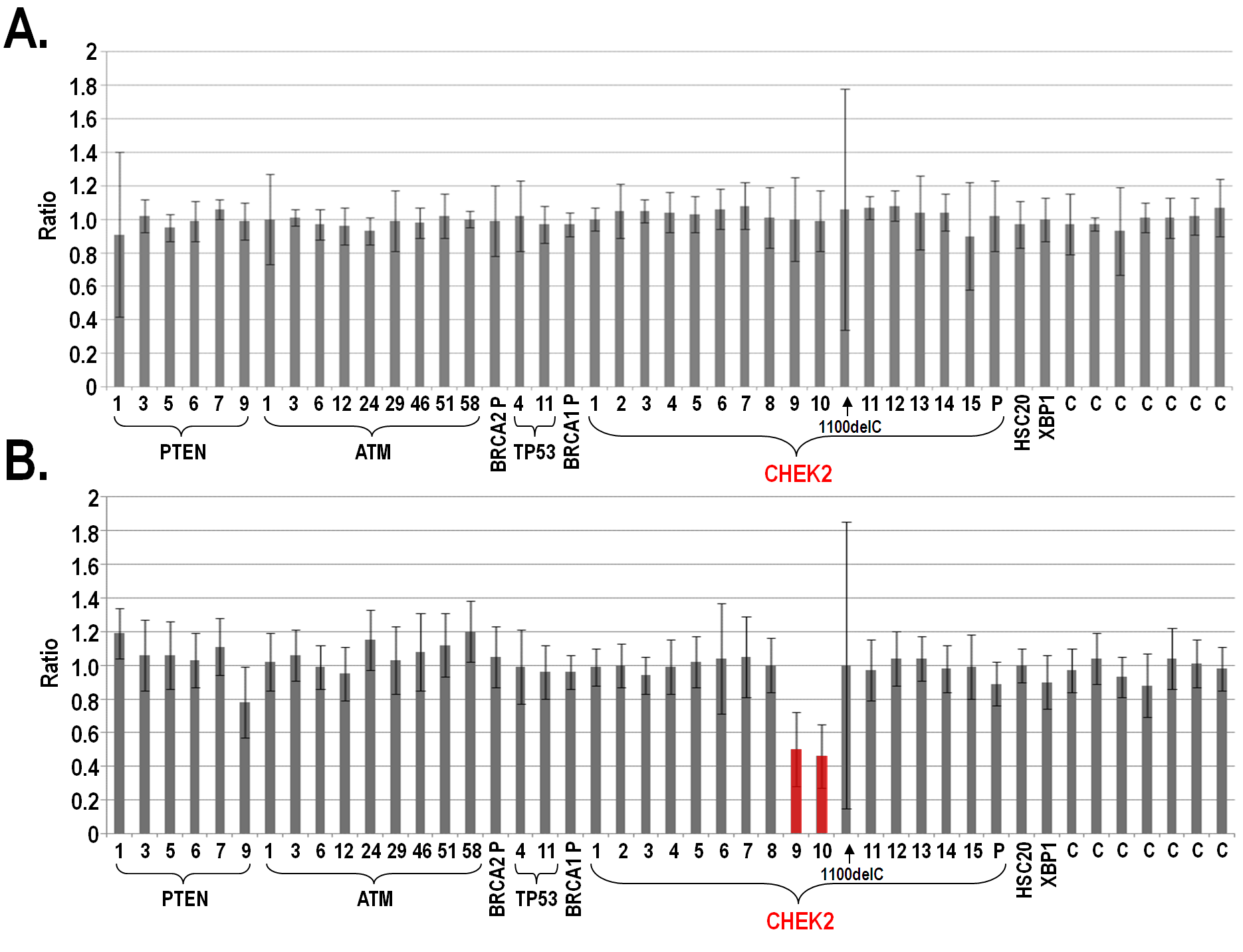

Supplement: S3 Fig — Results of the MLPA analysis using the Coffalyser software showing a sample with a wild-type CHEK2 sequence (A) and a sample carrying a large 5395 bp deletion (B) affecting the exons 8 and 9 (denominated in MLPA as exons 9 and 10, red bars). (TIF) [file pone.0140819.s003.tif]

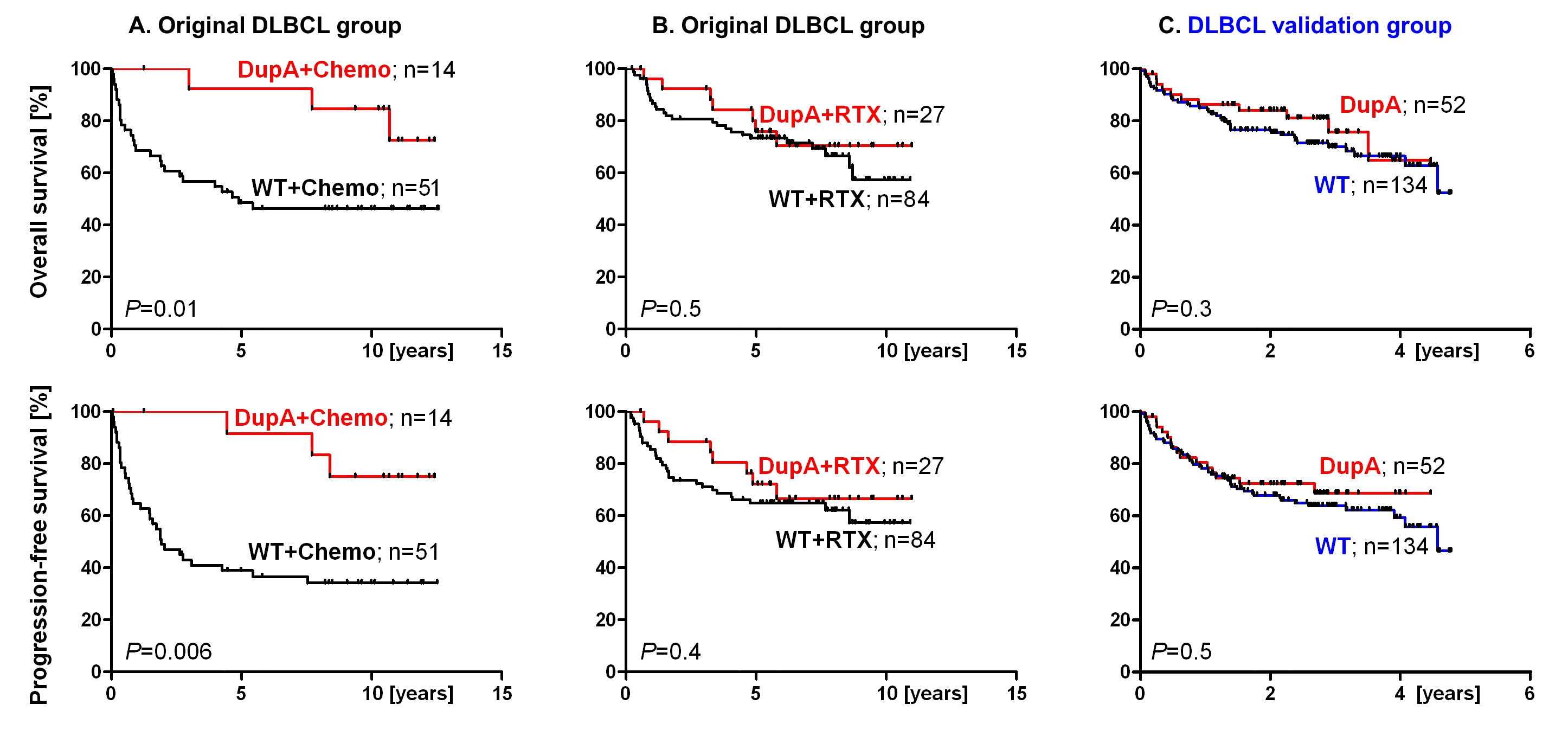

Supplement: S4 Fig — Panels show the OS and PFS in: A. patients treated only by conventional chemotherapy (HROS = 0.4; 95% CI 0.18–0.91; HRPFS = 0.4; 95% CI 0.17–0.74), B. patients treated by rituximab-based chemoimmunotherapy (RTX; HROS = 0.8; 95% CI 0.36–1.17; HRPFS = 0.7; 95% CI 0.36–1.50), and C. all patients from the DLBCL validation group (95% of patients treated with rituximab-based regimen; HROS = 0.8; 95% CI 0.40–1.39; HRPFS = 0.8; 95% CI 0.47–1.40). (TIF) [file pone.0140819.s004.tif]
