# Supplementary material for: Association of Germline CHEK2 Gene Variants with Risk and Prognosis of Non-Hodgkin Lymphoma
Source: PLoS One. 2015 Oct 27;10(10):e0140819. doi: 10.1371/journal.pone.0140819 (PMC4624763; doi:10.1371/journal.pone.0140819)
Supplement: S1 Table — (PDF) [file pone.0140819.s005.pdf]

S1 Table. Conditions for PCR amplification of CHEK2 coding sequence and following DHPLC analysis

CHEK2 coding exon	Primer				PCR reaction											DHPLC	
	Name	Sequence [5'-3']	Author[1]	Amplicon [bp]	Reaction volume [ul]	Type of enzyme[2]	Amount of enzyme per reaction [U]	Primer [pmol/ul]	dNTP's [mmol/ul] each	Mg^{2+} [mmol/ul]	DMSO [%]	Template DNA [ug]	Cycle[3]	Annealing [°C]	Cycles [N]	Oven [°C]	Time shift [min]
**0**	CHK32f	CTAAGTTCCGCTCTCCCTTC	This work	258	20	Gold	0.5	0.6	0.25	3	5	50	A	62-55	15+25	64.1	1.5
	CHK33r	CTTAAGATGGGATTCGAACCAC	This work														
**1**	CHK01f	AACTCACCTTTGTTGTTGGACA	Dufault 2004	441	25	Gold	0.6	0.6	0.25	3	5	50	A	62-55	15+25	57.0 + 61.0	0 + 1.5
	CHK02r	CAGAACCTTCCACCTGGTAATAC	This work														
**2-3**	CHEK11f	TCAACAGCCCTCTGATGCATG	This work	460	20	Gold	0.5	0.6	0.25	3	5	50	A	62-55	15+25	55.4	0
	CHEK15r	ACGCCCAGCAACTTACTCATC	This work														
**4**	CHK05f	ATCAGTGATCGCCTCTTGTG	Dufault 2004	365	20	Gold	0.5	0.6	0.25	3	5	50	A	62-55	15+25	53	0
	CHK06r	CAACACCCTGTCTCACAAAGA	Dufault 2004														
**5**	CHK07f	TCACTGTGTCCTCTGCAAAC	Dufault 2004	310	20	Gold	0.5	0.6	0.25	3	5	50	A	62-55	15+25	56.5	0
	CHK08r	TTGGGAAGTTATGAAGACGTGTTA	Dufault 2004														
**6**	CHK09f	CTCAGGCAGCCTTGAGTCAAC	Modified Dufault 2004	257	20	Gold	0.5	0.6	0.25	3	5	50	A	62-55	15+25	53.3	0
	CHK10r	CCACCACACCTGGCCAATATTATC	Modified Dufault 2004														
**7**	CHK11f	CTTGTGGTTTTCCTCTTGGGA	Modified Dufault 2004	213	20	Gold	0.5	0.6	0.25	3	5	50	A	62-55	15+25	54.5	0
	CHK12r	GATGAGAAAGGCAAGCCTACA	Dufault 2004														
**8**	CHK13f	ATTGTCTTCTGTCCAAGTGCG	Modified Dufault 2004	268	20	Gold	0.5	0.6	0.25	3	5	50	A	62-55	15+25	58.7	0
	CHK14r	CTCTTCTGAGTTTTAATCCACGGTC	This work														
**9**	CHK15f	AAGTATCTACTGCATGAATCTGAG	Modified Dufault 2004	301	20	Gold	0.5	0.6	0.25	3	5	50	A	62-55	15+25	56.4	0
	CHK16r	ATTCGAATCTGGATAAGAGCAG	Modified Dufault 2004														
**10-14**	CHK27f	CGACGGCCAGTCTCAAGAAGAGGACTGTCTT	Dufault 2004	9214	15	Expand	1.125	0.3	0.5	2.75 (buffer 2)	0	100	B	61	10+21	ND	ND
	CHK29r	GCTATGACCATGCACAAAGCCCAGGTTCCATC	Dufault 2004														
**10**	CHEK05f	TGGCAAGTTCAACATTATTCCC	Offit 2003	264	25	Gold	0.6	0.6	0.25	3	5	1ul of PCR product 10-14	C	54	35	56.3 + 58.3	0 + 1.5
	CHEK04r	ATTTGTGACTTCATCTAATCACCTCC	Offit 2003														
**11**	CHK19f	TGAGAATGCCACTTGATTTCTTT	Dufault 2004	217	20	Gold	0.5	0.6	0.25	3	5	1ul of PCR product 10-14	C	56	40	56.5	0
	CHK20r	GCACATACACATTTTAGCATACCA	Dufault 2004														
**12**	CHK21f	TTTATCCTTTTCACTGTGATTTGC	Dufault 2004	190	20	Gold	0.5	0.6	0.25	3	5	1ul of PCR product 10-14	C	55	40	59.5	0
	CHK22r	CATGTCTCTCAGGCAGCAG	Dufault 2004														
**13**	CHK23f	GGAGTTTATTATCCTTCAGACACAGC	Dufault 2004	182	20	Gold	0.5	0.6	0.25	3	5	1ul of PCR product 10-14	C	56	40	57	0
	CHK24r	AGCTCCTTAAGCCCAGACTACAT	Dufault 2004														
**14**	CHK30f	CACTTTACTGGAAGCATATTGAGG	This work	309	25	Gold	0.6	0.6	0.25	3	5	1ul of PCR product 10-14	C	56	40	54.5 + 61.5	0 + 3.4
	CHK26r	CATCAGTGACTGTGAAAAAGCAA	Dufault 2004														

Legend:

[1] Dufaul 2004: Dufault et al. Int J Cancer 2004;110(3):320-5

Offit 2003: Offit et al. BMC Med Genet 2003;4:1

[2] Expand: Expand Long Template PCR System (Roche)

Gold: AmpliTaq Gold (Life Technologies)

[3] Cycle A: 95°C 10 min; 15 cycles (95°C 30 s, 62°C - 0.5°C/cycle 30 s, 72°C 50 s) + 25 cycles (95°C 30 s, 55°C 30 s, 72°C 50 s); 72°C 10 min; 95°C 10 min, 60°C 5 min, 50°C 5 min

Cycle B: 94°C 2 min; 10 cycles (94°C 10 s, 61°C 30 s, 68°C 8 min) + 21 cycles (94°C 10 s, 61°C 30 s, 68°C 8 min + 20 s/cycle); 68°C 7 min

Cycle C: 95°C 10 min; 40 cycles (95°C 30 s, 54-56°C 30 s, 72°C 50 s); 72°C 10 min; 95°C 10 min, 60°C 5 min, 50°C 5 min
