# Supplementary material for: Association of Germline CHEK2 Gene Variants with Risk and Prognosis of Non-Hodgkin Lymphoma
Source: PLoS One. 2015 Oct 27;10(10):e0140819. doi: 10.1371/journal.pone.0140819 (PMC4624763; doi:10.1371/journal.pone.0140819)
Supplement: S2 Table — (PDF) [file pone.0140819.s006.pdf]

S2 Table. Predicted effects of the *CHEK2* missense variants identified in NHL patients in this study using software prediction.

		Align GVGD		SIFT		PolyPhen2		Mutation Taster
**Protein change**	**Exon**	**GV/GD**	**Class**	**Score**	**Prediction**	**Probability**	**Prediction**	**Prediction**
p.E64K	3	44.60/56.87	C15	0.54	tolerated	0.170	benign	disease causing
p.I157T	3	50.29/69.84	C15	0.07	tolerated	0.385	benign	disease causing
p.R180C	3	86.39/137.37	C25	0.01	damaging	0.807	possibly damaging	disease causing
p.R181H	3	229.27/0	C0	0.55	tolerated	0.058	benign	polymorphism
p.E239K	5	29.27/51.63	C15	0.19	tolerated	0.136	benign	disease causing
p.S356L	9	135.42/14.3	C0	0.21	tolerated	0.991	probably damaging	disease causing
p.T401A	10	134.86/1.01	C0	0.91	tolerated	0.004	benign	disease causing
p.N446D	11	129.03/22.66	C0	0.77	tolerated	0.003	benign	polymorphism
p.R474H	12	0/28.82	C25	0.00	damaging	1.000	probably damaging	disease causing

Align GVGD: http://agvgd.iarc.fr/agvgd_input.php

SIFT: http://sift.jcvi.org/www/SIFT_enst_submit.html

PolyPhen-2: http://genetics.bwh.harvard.edu/pph2/index.shtml

Mutation Taster: http://www.mutationtaster.org/
