# Supplementary material for: Association of Germline CHEK2 Gene Variants with Risk and Prognosis of Non-Hodgkin Lymphoma
Source: PLoS One. 2015 Oct 27;10(10):e0140819. doi: 10.1371/journal.pone.0140819 (PMC4624763; doi:10.1371/journal.pone.0140819)
Supplement: S3 Table — (PDF) [file pone.0140819.s007.pdf]

S3 Table. Risk of NHL subtypes development in carriers of mutations modifying CHK2 protein structure and c.319+43dupA polymorphism.

NHL subtype	NHL patients			Controls (*n*=450)		OR (95% CI)	*P* value
	n	**with variant**	**%**	**with variant**	**%**		
***CHEK2* alterations affecting coding sequence**							
DLBCL	180	11	6.1	12	2.7	2.35 (1.02-5.43)	0.06
FL	71	5	7.0	12	2.7	3.33 (1.21–9.19)	**0.03**
MCL	19	3	15.8	12	2.7	6.77 (1.74–26.37)	**0.02**
B-SLL	11	3	27.3	12	2.7	13.53 (3.19-57.45)	**0.004**
**c.319+43dupA (A/AA+AA/AA)**							
DLBCL	180	42	23.3	139	31.2	0.67 (0.45–1.00)	0.05
FL	71	14	19.7	139	31.2	0.54 (0.29–1.00)	0.05

Abbreviations:

DLBCL diffuse large B-cell lymphoma; FL - follicular lymphoma; MCL - mantle cell lymphoma; B-SLL - B-small lymphocytic lymphoma
