# Supplementary material for: Association of Germline CHEK2 Gene Variants with Risk and Prognosis of Non-Hodgkin Lymphoma
Source: PLoS One. 2015 Oct 27;10(10):e0140819. doi: 10.1371/journal.pone.0140819 (PMC4624763; doi:10.1371/journal.pone.0140819)
Supplement: S4 Table — (PDF) [file pone.0140819.s008.pdf]

S4 Table. Comparison of therapy type between c.319+43dupA carriers and non-carriers (original group of DLBCL patients) and between two analyzed DLBCL groups.

**Type of chemotherapy**		**DLBCL, original group**				***P*-value**	**Any genotype, DLBCL original group**		**Any genotype DLBCL validation group**		***P*-value**
		**DupA carriers**		**Without DupA**							
		n	**%**	***n***	**%**		***n***	**%**	***n***	**%**	
CHOP	with R	13	31.7	54	40.0	0.80	67	38.1	111	59.7	**<0.001**
	All	19	46.3	86	63.7	0.07	105	59.7	111	59.7	1.00
megaCHOP	with R	6	14.6	11	8.1	0.60	17	9.7	13	7.0*	0.10
	All	8	19.5	13	9.6	0.10	21	11.9	13	7.0*	0.10
COP	with R	1	2.4	3	2.2	1.00	4	2.3	10	5.4	**0.02**
	All	1	2.4	7	5.2	0.70	8	4.5	10	5.4	0.80
Other	with R	7	17.0	16	11.9	1.00	23	13.1	42	22.6	**0.01**
	All	13	31.7	29	21.5	0.20	42	23.9	52	28.0	0.40
CHOP-like[1]	with R	20	48.8	68	50.4	0.70	88	50.0	113	60.8	**<0.001**
	All	28	68.3	102	75.6	0.40	130	73.9	113	60.8	**0.01**
Any chemotherapy[2]	with R	27	65.9	84	62.2	0.70	111	63.1	176	94.6	**<0.001**
	All	41	100.0	135	100.0		176	100.0	186	100.0	

* include 12 patients treated within R-megaCHOP-ESHAP-BEAM trial (as described in details in Pytlik R, et al. (2015) Leuk Lymphoma 56:57-64.)

[1] CHOP-like includes: CHOP, megaCHOP and CHOP14

[2] Other includes: CHOP14, ESHAP, ICE, MTX based regimens or ASCT

Abbreviations:

DupA - c.319+43dupA

R – rituximab
